# Klebsiella Pneumoniae Brain Abscess in a Patient With Chronic Lymphocytic Leukemia

**DOI:** 10.7759/cureus.32479

**Published:** 2022-12-13

**Authors:** Anisha Rajavel, Waleed Sadiq, Madeeha Subhan Waleed, Dany El-Sayegh

**Affiliations:** 1 Internal Medicine, Staten Island University Hospital, Staten Island, USA; 2 Internal Medicine, Lower Bucks Hospital, Bristol, USA; 3 Pulmonary Critical Care, Staten Island University Hospital, Staten Island, USA

**Keywords:** immunocompromised, tonsillar abscess, critical care, prostate cancer, ibrutinib, hypogammaglobulinemia, chronic lymphocytic leukemia (cll), brain abscess, meningitis, klebsiella pneumoniae (kp)

## Abstract

*Klebsiella Pneumoniae* (*K. pneumoniae*) is a common nosocomial pathogen. However, *Klebsiella-*associated meningitis and brain abscess formation are extremely rare in the United States. We present a case of a 73-year-old male who initially presented for a tonsillar abscess of unknown etiology. While awaiting an abscess biopsy, the patient underwent molar extraction for chronic periodontitis and decay. The patient subsequently developed *K. pneumoniae* bacteremia and meningitis. As he clinically declined, repeat imaging revealed a brain abscess with eventual hemorrhagic transformation. Notably, the patient had underlying hypogammaglobulinemia from chronic lymphocytic leukemia (CLL), which we believe contributed to the invasive disease. Given the global spread of virulent strains of *Klebsiella* (such as hypervirulent or hypermucoviscous *K. pneumoniae*), clinicians must bear this pathogen in mind while treating critically ill and immunocompromised patients.

## Introduction

*Klebsiella pneumoniae* (*K. pneumoniae*) is a gram-negative bacteria that is commonly encountered in the healthcare setting and responsible for almost 10% of all hospital-acquired infections in the United States [1,2]. *K. pneumoniae* typically cause pneumonia and urinary tract infections, although a more invasive disease can occur in immunocompromised patients [2]. Community-acquired *Klebsiella-*associated meningitis is rare in the United States, especially in the absence of neurosurgical history [3]. More virulent and drug-resistant variants of* K. pneumoniae* (such as hypermucoviscous *Klebsiella*) from other countries have been reported in the United States [4]. These strains have been associated with more invasive diseases, such as meningitis, endophthalmitis, and liver abscesses [4]. The sources and routes of entry associated with invasive *Klebsiella* infection are not fully understood [4]. We report a rare case of severe *Klebsiella*-associated meningitis and brain abscesses in a patient with underlying hypogammaglobulinemia. 

## Case presentation

**Figure 1:**
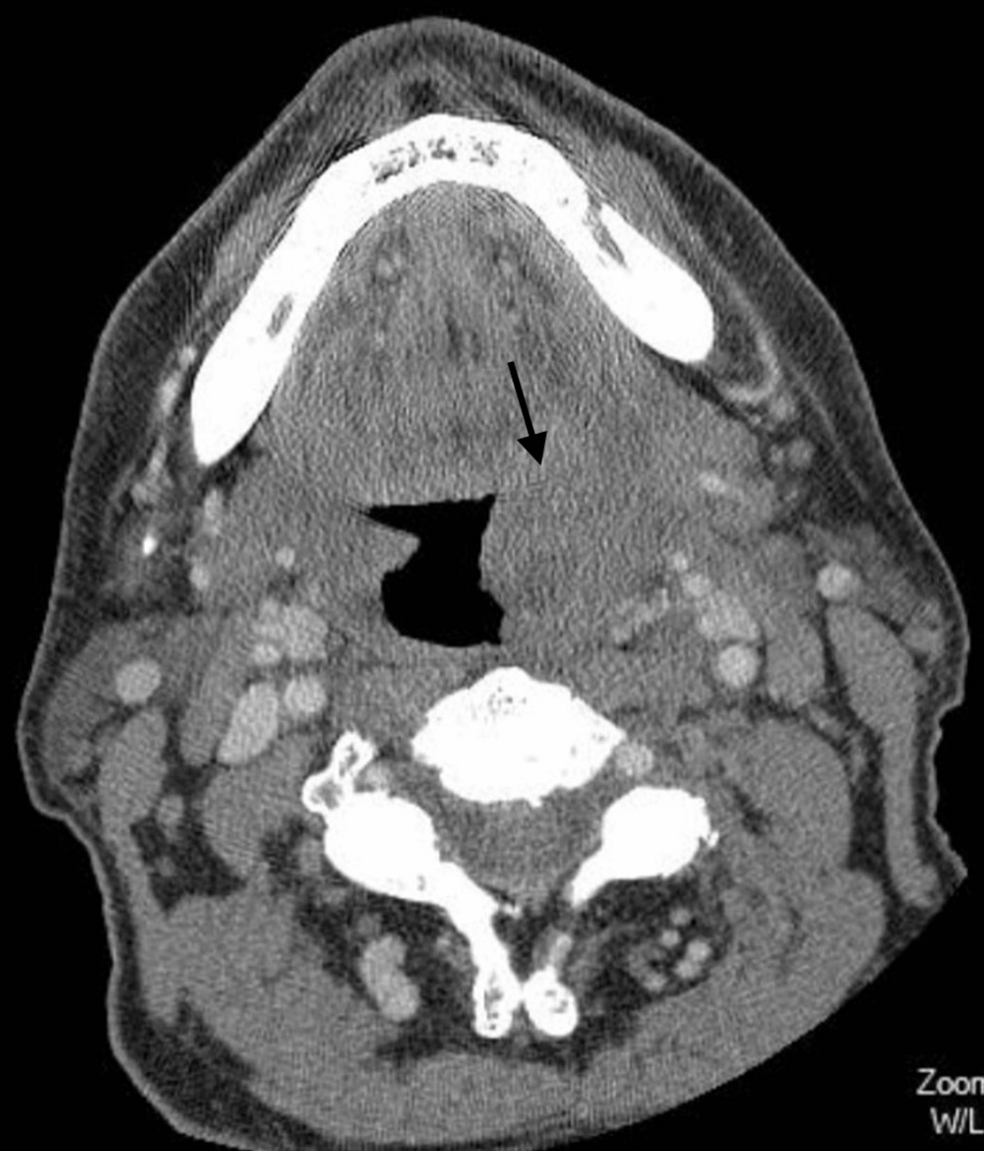
Initial CT scan with IV contrast of left palatine tonsillar mass before undergoing outpatient antibiotic treatment.

A 73-year-old male with a medical history of chronic lymphocytic leukemia (CLL) diagnosed three months before admission (being treated with ibrutinib) and prostate cancer (untreated, with surveillance of prostate-specific antigen only) presented to the hospital for dysphagia and throat pain. Symptoms had slowly progressed over five weeks without a history of trauma or instrumentation to the area. The patient denied any fevers and reported only mild throat pain and an inability to fully swallow food. He had no cognitive impairment and was fully functional and ambulatory on admission. Upon admission to the emergency department, he had a maxillofacial computed tomography (CT) scan with intravenous (IV) contrast, which revealed asymmetric enlargement of the left palatine tonsil, with prominent bilateral lingual tonsils and effacement of the left glossotonsillar sulcus (Figure 1). The patient was discharged within one day and given a 10-day course of antibiotics to complete outpatient (oral amoxicillin-clavulanate). 

However, his symptoms persisted, and the patient returned to the hospital approximately three weeks later. On repeat admission (day one), he had another CT scan of the head and neck, which showed fullness in the left palatine tonsil, and appeared similar to the prior imaging. Given the persistence of the mass, the radiologist suspected the lesion may be malignant. The patient was given one dose of 3g IV ampicillin-sulbactam in the emergency department while awaiting admission but was then monitored off antibiotics. Dexamethasone IV 10mg daily was initiated for swelling, and the patient received a total of 14 days of steroids. Symptoms persisted despite steroids, and an inpatient biopsy of the left tonsil was subsequently planned.

**Figure 2:**
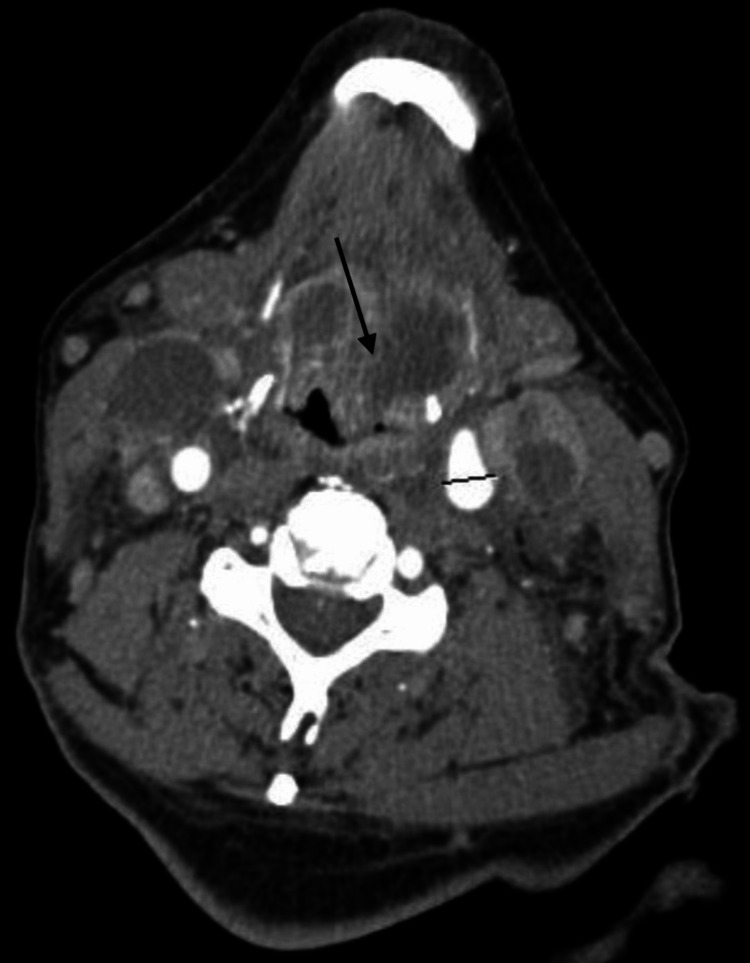
Bilateral, necrotic cervical lymph nodes on CT angiogram with IV contrast of neck. Necrotic tissue was noted in sublingual space, with some impingement of hypopharynx (arrow) before tracheostomy placement.

On day five, while awaiting a biopsy of the tonsillar mass, the patient complained of chronic dental pain. He was evaluated by the dental team and underwent extraction of molars # 18 and 19 for periodontitis and chronic, recurrent decay. On day six, he underwent a bedside biopsy of the left tonsil by an otolaryngologist using cupped forceps. The biopsy showed no evidence of new malignancy but did show acute inflammatory changes with rare cells consistent with the known diagnosis of CLL. On day seven, he became progressively lethargic with high fevers. Initial suspicion of a stroke prompted a CT scan without contrast of the head and a CT angiogram with IV contrast of the head and neck, which revealed no acute stroke or hemorrhage. However, imaging showed several bilateral, necrotic cervical lymph nodes and necrotic tissue in the sublingual space with obstruction of the hypopharynx (Figure 2). On day eight, the patient had a transthoracic echocardiogram (TTE), which did not reveal any valvular lesions. His respiratory status declined on day eight, and he required intubation for airway protection due to obtundation. Intubation was unsuccessful due to airway compromise, and he underwent emergent surgical airway placement (tracheostomy). Empiric antimicrobials (IV meropenem, IV linezolid, and IV acyclovir) for suspected meningitis were initiated.

Additionally, on day eight, blood cultures that were drawn on day seven of hospitalization were found to be positive for *Klebsiella pneumoniae.* On day nine, a lumbar puncture was performed, which revealed purulent and cloudy cerebrospinal fluid (CSF). CSF protein was elevated at 484 mg/dL, glucose was 64 mg/dL, peripheral serum glucose was 222 mg/dL, and total nucleated cell count was 3302 cells/µL with 80% neutrophils. The opening pressure was approximately 20 mmHg. CSF gram staining did not show any organisms, but CSF culture revealed *Klebsiella pneumoniae*. Antibiotics were narrowed to IV ceftriaxone and IV ciprofloxacin, tailored to the resulting sensitivities. Broad PCR testing of the CSF for Lyme disease, Epstein-Barr Virus, Herpes Simplex, and West Nile Virus was otherwise negative. The patient was monitored on antibiotics and steroids alone, and no further biopsies, incisions, or drainage of the mass were performed. On day nine, the hospital course was further complicated by new-onset atrial fibrillation. The patient was started on an infusion of IV Cardizem and was switched to oral (PO) Cardizem once the heart rate was controlled. Therapeutic anticoagulation using low-molecular-weight heparin was initiated. 

**Figure 3:**
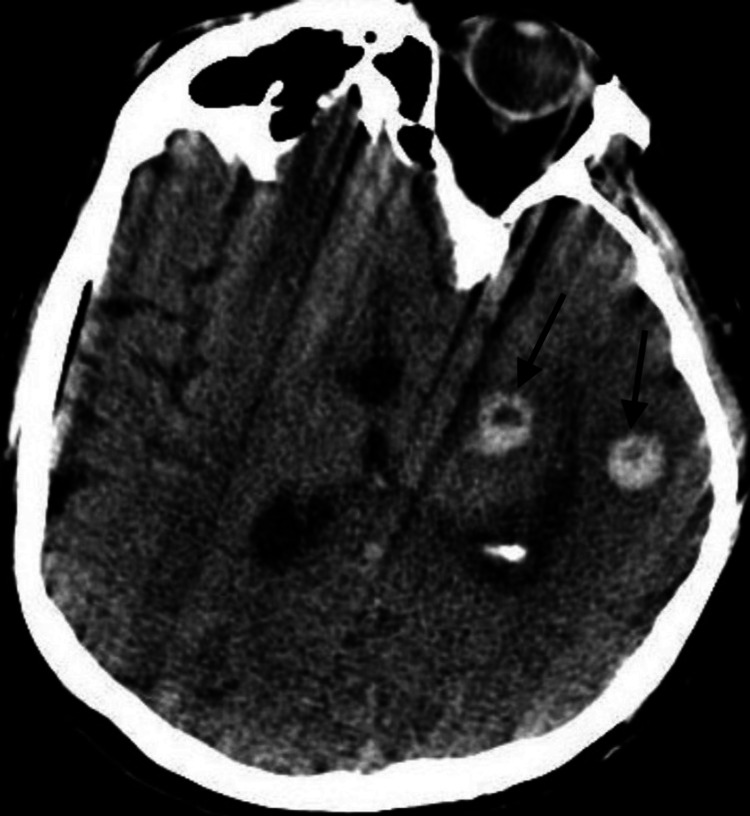
CT imaging without contrast revealed two ring-enhancing hyperdense lesions of 1.5 cm and 1.2 cm found in the left basal ganglia and left temporal lobe, respectively.

**Figure 4:**
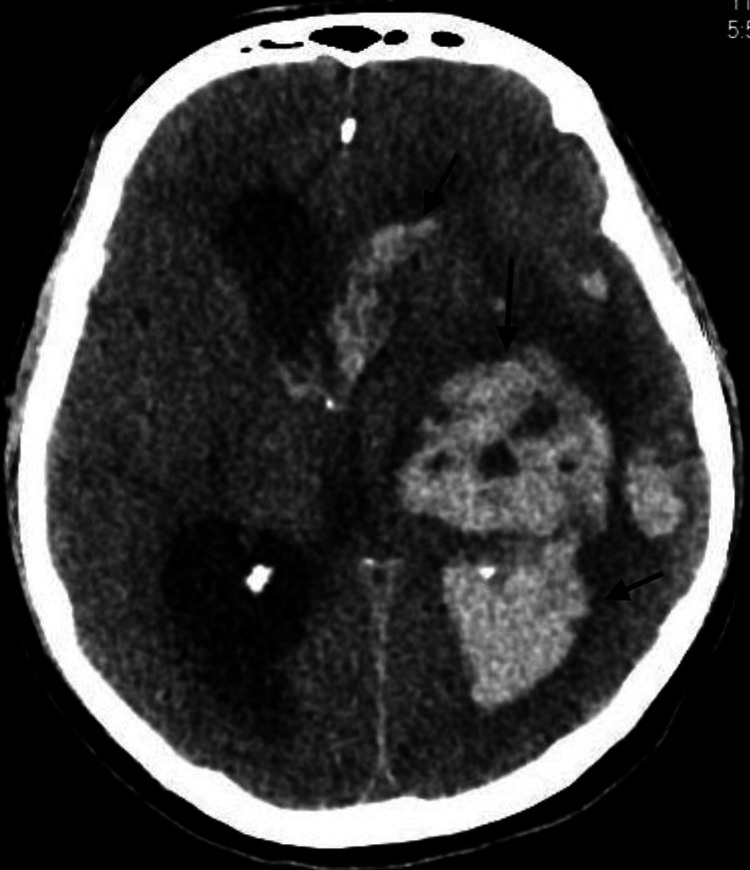
Hemorrhagic transformation of abscesses on CT imaging without contrast, following a drastic change in the neurological exam.

Given the patient’s history of CLL, immunoglobulin levels were quantified. Total IgG levels revealed hypogammaglobulinemia (total IgG 198 mg/dL, IgA 43 mg/dL, and IgM <10 mg/dL). We administered one dose of intravenous immunoglobulin (IVIg) at a dose of 500 milligrams/kilogram (mg/kg) on day 14, after which the patient remained on the ventilator but showed some clinical improvement in mental status. On day 15, a repeat CT scan without contrast of the head was performed to rule out increased intracranial pressure (ICP) before a repeat lumbar puncture. Despite modest improvement in mental status, including following hand-grip commands and tracking with eyes while off of sedation, a CT scan revealed two ring-enhancing hyperdense lesions of 1.5cm and 1.2cm in the left basal ganglia and left temporal lobe, respectively (Figure 3). Additional plans for a transesophageal echocardiogram (TEE) to rule out endocarditis were made, although the patient clinically declined before the procedure. A neurosurgeon was consulted on day 16, who recommended monitoring on antibiotics alone without surgical intervention. On day 18, the patient was found to have fixed, dilated pupils. CT scan without contrast of the head revealed a massive hemorrhagic transformation of the abscesses into the lateral, third, and fourth ventricles (Figure 4). Notably, his platelet count on day 17 and day 18 ranged from 75 to 135, and he was receiving therapeutic low-molecular-weight heparin for atrial fibrillation. There was a 1.2 cm midline shift and changes suggestive of increased ICP and imminent uncal herniation. The patient’s family decided to pursue palliative care only.

## Discussion

*Klebsiella pneumoniae* is a common culprit of hospital-acquired infections and is responsible for nosocomial urinary tract infections, pneumonia, and bacteremia [2]. Despite the ubiquitous nature of this organism, *K. pneumoniae-*associated meningitis is rare in the United States in the absence of neurosurgical risk factors [3]. Given recent global reports of Hypervirulent (or Hypermucoviscous) *K. pneumoniae, *we must study the various clinical presentations of this organism. Because our hospital does not test for hypermucoviscous *K. pneumoniae*, we do not know whether our patient was infected with this strain. Nevertheless, the ubiquitous nature of conventional strains of *Klebsiella* in hospitals should prompt clinicians to keep this pathogen on the differential diagnosis for critically ill patients [4]. We report a case of a previously functional patient with hypogammaglobulinemia due to CLL, who became critically ill of *K. pneumoniae* meningitis and brain abscesses. 

Immunocompromised patients and those with underlying malignancies are traditionally at higher risk of *K. pneumoniae* bacteremia [1,2]. Our patient presented with a history of CLL on ibrutinib treatment, as well as untreated prostate cancer. Although he did not exhibit neutropenia during the hospitalization, he was found to have hypogammaglobulinemia from the CLL. Other risk factors for* K. pneumoniae *bacteremia include diabetes mellitus and liver disease, which our patient did not have [1]. ICU care and underlying hematological malignancies have also been described as risk factors for *Klebsiella* bacteremia, both of which were present in our patient [1]. Although the incidence of *Klebsiella*-associated meningitis remains low in the United States, we propose that clinicians caring for cancer patients, and other immunocompromised patients, maintain a high suspicion of this organism.

Identifying a clear nidus of infection was challenging in our case. Most cases of* K. pneumoniae* meningitis occur in neurosurgical patients, and community acquisition is rare, especially in the United States [3]. We were able to rule out urinary tract infection and pneumonia as primary causes for meningitis following a urinalysis that was unrevealing for infection and multiple chest radiographs without evidence of pulmonary involvement. The dental extraction of molars #18 and #19 appeared to have a clear chronologic association with meningeal seeding, as the patient’s mental status deteriorated 48 hours following the procedure. Biopsy of the left tonsillar gland took place one day after dental extraction and may also have been responsible for the spread to the central nervous system (CNS). There have been case reports of meningitis following peritonsillar abscesses and tonsillectomies, although these are largely found in the pediatric population and are not restricted to *K. pneumoniae* as the causative organism [5,6]. Infection from the tonsils can spread hematogenously in a retrograde pattern through the jugular vein [5,6]. Seeding of the perineural sheaths of extracranial nerves during a biopsy can also transmit the infection to the CNS [5,6]. Immunocompromised patients may be at higher risk for these rarer routes of infection. Further investigations should be pursued to study the role of prophylactic antibiotics in immunocompromised populations before undergoing non-invasive procedures such as dental extractions or gland biopsies.

Hypervirulent (Hypermucoviscous) *Klebsiella* is a highly-infectious variant of *K. pneumoniae* that has been reported throughout the Asian Pacific Rim, although there have been case reports of this variant throughout the world, including in the United States [4]. This strain has been traditionally identified by the "string test," in which an inoculation instrument is used to create a sticky "string" of greater than 5 mm, although the test is rarely performed [4]. Our hospital does not test for these strains because it does not alter the antibiotic susceptibilities of the organism, and treatment typically remains unchanged [4]. Other drug-resistant variants, such as New Delhi Metallo-β-lactamase- containing *Klebsiella* (NDM-1), have also been reported in the United States, possibly spread via medical tourism [4]. Our hospital does routine tests for drug-resistant variants, and our patient had a strain that was sensitive to commonly used third-generation cephalosporins (ceftriaxone). Given the global spread of hypervirulent and drug-resistant variants of *K. pneumoniae* and the potential for conventional *Klebsiella* strains to cause severe infection in immunocompromised populations, we maintain that clinicians have a low threshold for CSF sampling in high-risk patients. 

## Conclusions

Community-acquired meningitis and brain abscesses from *K. pneumoniae* are rare in the United States, with very few reported cases. Despite this, clinicians should maintain a suspicion of *Klebsiella* in patients exhibiting signs of meningitis due to the global spread of hypervirulent (hypermucoviscous) and drug-resistant strains of *K. pneumoniae*. Furthermore, even conventional strains of ​​​​​​​*Klebsiella* alone can cause catastrophic infection in immunocompromised patients. In our reported patient, meningitis and cranial disease could have developed from retrograde venous spread through the jugular veins or seeding of perineural sheaths following the tonsillar biopsy. Underlying hypogammaglobulinemia from CLL may have further facilitated this infection. Further studies should be conducted to study routes of entry in invasive *Klebsiella* infection and the role of prophylactic antibiotics in immunocompromised patients undergoing semi-invasive procedures. We further conclude that suspicion of ​​​​​​​*Klebsiella* should remain high in immunocompromised and critically ill populations. 
